# Fracture Properties of Polystyrene Aggregate Concrete after Exposure to High Temperatures

**DOI:** 10.3390/ma9080630

**Published:** 2016-07-28

**Authors:** Waiching Tang, Hongzhi Cui, Soheil Tahmasbi

**Affiliations:** 1 School of Architecture and Built Environment, The University of Newcastle, Callaghan NSW 2308, Australia; Soheil.tahmasbi@uon.edu.au; 2 Shenzhen Durability Center for Civil Engineering, Shenzhen University, Shenzhen 518060, Guangdong, China; h.z.cui@szu.edu.cn

**Keywords:** polystyrene aggregate concrete, fracture properties, high temperature

## Abstract

This paper mainly reports an experimental investigation on the residual mechanical and fracture properties of polystyrene aggregate concrete (PAC) after exposure to high temperatures up to 800 degrees Celsius. The fracture properties namely, the critical stress intensity factor ($K_{IC}^{S}$), the critical crack tip opening displacement (*CTOD_C_*) for the Two-Parameter Model, and the fracture energy (*G_F_*) for the Fictitious Crack Model were examined using the three-point bending notched beam test, according to the RILEM recommendations. The effects of polystyrene aggregate (PA) content and temperature levels on the fracture and mechanical properties of concrete were investigated. The results showed that the mechanical properties of PAC significantly decreased with increase in temperature level and the extent of which depended on the PA content in the mixture. However, at a very high temperature of 800 °C, all samples showed 80 percent reduction in modulus of elasticity compared to room temperature, regardless of the level of PA content. Fracture properties of control concrete (C) and PAC were influenced by temperature in a similar manner. Increasing temperature from 25 °C to 500 °C caused almost 50% reduction of the fracture energy for all samples while 30% increase in fracture energy was occurred when the temperature increased from 500 °C to 800 °C. It was found that adding more PA content in the mixture lead to a more ductile behaviour of concrete.

## 1. Introduction

The need for lightweight concretes is rapidly increasing worldwide due to their benefits (e.g., lower dead weight and lower handling cost) over Normal Concretes (NC). Among the various types of proposed lightweight concretes, Polystyrene Aggregate Concrete (PAC) is interesting because they can be tailored to suit specific needs by changing some of their constituents’ properties such as the bead size and volume fraction of polystyrene. In addition, this concrete can be fabricated in construction sites, which is a huge advantage against materials like autoclaved cellular concrete. PAC is a lightweight concrete with a wide range of densities from 1000 to 2000 kg/m^3^ which can be produced by partially replacing coarse aggregate in the reference (normal weight) concrete mixtures with equal volume of the chemically coated polystyrene beads. Light weight, good thermal properties, excellent sound insulation, improved durability and environmental friendliness (polystyrene aggregate can be produced from recycled material) [1,2,3] are some of the advantages of PAC making them a good choice for both structural and non-structural applications depending upon the amount of expanded polystyrene aggregate used. Previously, several studies were conducted on mix details, strength properties, drying shrinkage, creep, compaction & finishing etc. of the PAC [3,4,5,6,7]. However, these studies have been essentially related to PAC of lower strength. To extend the use of PAC to meet both structural and functional requirements, a series of PAC of 1410–2100 kg/m^3^ densities with corresponding strengths of about 17 MPa minimum [8] was designed and the results on their mechanical and time-dependent properties were reported in the previous papers [9,10]. The focus of this paper is to report the mechanical and fracture properties of PAC after exposure to high temperatures.

Fracture energy (*G_F_*_)_ is one of the most important material properties to assess the toughness and brittleness of concrete. The larger the value of *G_F_*, the less brittle or the more work is necessary to cause failure. Although a considerable amount of research work has been done on PAC, the information about its fracture properties, in particular the focus on fracture energy under high temperature is very limited. Trussoni et al. [11] measured the fracture energy of PAC and normal weight concrete (NWC) via the three-point-bend test. Their results shown that PAC changes its failure mode in compression by exhibiting a more ductile dissipation of load during failure. They further indicated that polystyrene aggregate concrete is capable to absorb more energy and maintain load after reaching peak load. As a result, the fracture energy (*G_F_*) for PAC is considered to be higher than that for NC, as more energy is absorbed when creating more fracture surface in PAC. Sabaa [12] reported the polystyrene aggregate particles are capable to act as energy absorbing component in the concrete system, thus exhibiting a more ductile behaviour during failure. Previous limited studies on PAC has merely focused on fracture energy under room temperature conditions, however the fracture energy of normal and other lightweight concretes generally decreases with temperature as reported in the literature [13,14,15]. Therefore, it’s of great interest to study the effect of high temperature on the fracture energy and brittleness of PAC.

## 2. Theoretical Background

In the Fictitious Crack Model (FCM), the main parameters are the tensile strength (*f_t_*) and the fracture energy (*G_F_*). According to the RILEM Recommendation [16], the fracture energy is evaluated from the three-point bending test on a notched beam using the following equation:

(1) $$G_{F}=(A+mg\delta_{0})/b(d-a_{0})$$

where *A* is the area under the load-deflection curve and *δ*_0_ is the deflection when the load capacity is zero. *g*, *m*, *b*, *d* and *a*_0_, are gravity factor, mass, width, height and notch depth of the beam. To describe the brittleness of a material, the characteristic length has been suggested as:

(2) $$I_{ch}=EG_{F}/{\left(f_{t}\right)}^{2}$$

**Figure 1:**
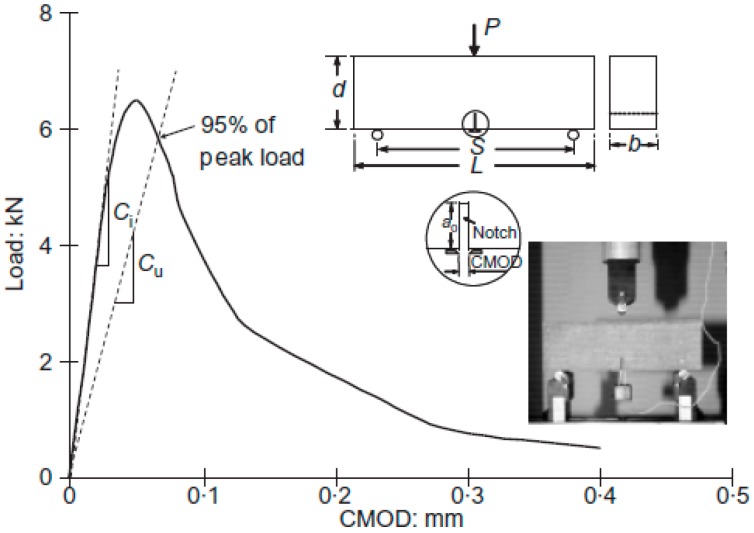
Typical experimental load-CMOD plot with testing configuration and geometry of specimen (*C_i_* and *C_u_* are calculated from the load-CMOD curve in compliance with RILEM).

In the Two-Parameter Model (TPM) by Jenq and Shah [17] the critical stress intensity factor ($K_{IC}^{S}$) and critical crack tip opening displacement (*CTOD_C_*) are also determined from the three-point bending test on a notched beam in accordance with the RILEM recommendation. Firstly, the modulus of elasticity can be obtained from the initial compliance of the load-CMOD curve by using

(3) $$E=6Sa_{0}V_{1}(a_{0}/d)/(C_{i}bd^{2})$$

where *C_i_*, *S*, *b*, *d* and *a*_0_, are the initial compliance, span, thickness, height and initial notch depth respectively as shown in Figure 1. *V*_1_ is a geometric function expressed as

(4) $$V_{1}(\alpha )=0.76-2.28\alpha +3.87\alpha^{2}-2.04\alpha^{3}+0.66/{\left(1-\alpha \right)}^{2}$$

where *α* = *a*_0_/*d*, in which the thickness of holder of clip gauge was neglected. The critical stress intensity factor ($K_{IC}^{S}$) is then calculated from:

(5) KICS=3(Pmax+0.5WoS/L)S(πac)1/2 F (ac/d)/2bd2

where *W_o_* and *L* are the self-weight and length of the beam respectively. *P_max_* is the measured maximum load. *F* is a geometric function expressed as where *γ* = *a_c_*/*d*, in which *a_c_* is the critical effective crack length and determined by iteration from

(6) $$F(\gamma )=[1.99-\gamma (1-\gamma )(2.15-3.93\gamma +2.7\gamma^{2})]/\sqrt{\pi }(1+2\gamma ){\left(1-\gamma \right)}^{3/2}$$

(7) $$a_{c}=EC_{u}bd^{2}/6SV_{1}(\gamma )$$

where *C_u_* is the unloading compliance at 95% of the peak load which can be approximately calculated by assuming the unloading path will return to the origin [18] as shown in Figure 1. The critical crack tip opening displacement can be obtained from:

(8) $$CTOD_{C}=6P_{\max}Sa_{c}V_{1}(a_{c}/d)V_{2}(a_{c}/d,a_{0}/a_{c})/(Ebd^{2})$$

where

(9) $$V_{2}(\gamma ,\beta )={\left[{\left(1-\beta \right)}^{2}+(1.081-1.149\gamma )(\beta -\beta^{2})\right]}^{1/2}$$

in which *γ* = *a_c_*/*d* and *β* = *a*_0_/*a_c_*. The critical strain energy release rate is related to the critical stress intensity factor by:

(10) $$G_{IC}^{S}={\left(K_{IC}^{S}\right)}^{2}/E$$

A material length is also introduced as

(11) $$Q={\left(E\times CTOD_{C}/K_{IC}^{S}\right)}^{2}$$

which is proportional to the size of the fracture process zone and can be used to describe the brittleness of a material.

## 3. Materials and Methods

### 3.1. Materials

**Figure 2:**
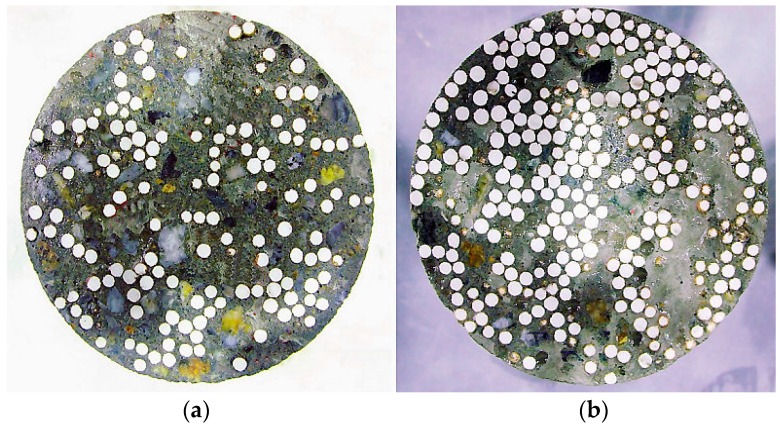
PA distribution of (**a**) Mix ‘PA25’ and (**b**) Mix ‘PA50’.

In this research, a control concrete (C) and two PAC mixes (PA25 and PA50) were studied. These two PAC mixes were proportioned by replacing 25 and 50 percent of the normal coarse aggregate from the control concrete with an equal bulk volume of polystyrene aggregate respectively. Figure 2 shows the even distribution of polystyrene aggregate within both PAC mixes. The polystyrene aggregate was made from small polystyrene beads, coated with a non-toxic and patented chemical compound that was supplied by BST (East Asia) Ltd. (Hong Kong, China). The purpose of coating is to overcome the hydrophobicity of polystyrene beads and the proneness to segregation in polystyrene concrete mixes. 

The mean diameter and bulk density of the single-size polystyrene aggregate particles were approximately 4 mm and 24 kg/m^3^, respectively. The materials used in this study were Ordinary Portland cement (OPC) conforming to BS EN 197-1:2001 [19], river sand with a fineness modulus of 2.75, and crushed granite with a nominal size of 10 mm. Table 1 shows the mix details used in this investigation. According to the findings of previous paper [9], the entrapped air content increased (up to 16%) when the 10 mm normal coarse aggregate was increasingly replaced with polystyrene aggregate (4 mm) to produce PAC mixes. Therefore, it can be stated that PAC mixes are consisted of low modulus polystyrene aggregate and high modulus 10 mm coarse aggregate as well as a high amount of large sized air voids, randomly distributed in concrete. The concrete with polystyrene aggregate was mixed, cast, demoulded and cured for 28 days in a controlled environment, similar to that of the control concrete.

### 3.2. Heating Profile

The residual properties of concrete specimens after exposure to four temperature levels (150, 400, 500 and 800 °C) were investigated. Heating of specimens in a furnace was performed at a heating rate of 5 °C/min up to the desired temperatures. The hot specimens were then allowed to cool down naturally in room temperature and followed by the mechanical and fracture tests.

### 3.3. Specimen Preparation and Testing

Standard concrete specimens were cast to study the strength properties (compressive strength, *f_c_*, tensile strength, *f_t_* and elastic modulus, *E*) according to the BS standards [20,21,22]. The notched concrete beams with the dimensions 75 × 75 × 250 mm^3^ (see Figure 1) were used to study the residual fracture energy (*G_F_*) according to RILEM recommendations [16]. The beams were notched centrally to 20 mm depth using a 2 mm diamond saw. A beam length of 250 mm rather than the length of 840 mm recommended by RILEM [16] was used to suit the internal dimensions of the furnace. Results of Brokenshire and Barr [23], and Zhou and Balendran [24] for normal temperature conditions have shown that there was no appreciable difference in the measured fracture energy due to such a size change. All the samples were kept in a controlled room at 25 °C and 60 percent RH prior to heating. The bending tests were conducted using a servo-controlled MTS universal testing machine. During the test, the crack mouth opening displacement (CMOD) was measured using a clip gauge clipped to the bottom of the beam and held in position by two steel knife edges glued to the specimen. The displacement rate was controlled at a suitable rate until the specimens failed. The critical stress intensity factor ($K_{IC}^{S}$) and critical crack tip opening displacement (*CTOD_C_*) in the Two-Parameter Model were evaluated from the load-CMOD curves which were measured from the three-point bending tests on notched beams, in accordance with the RILEM Recommendation [18].

## 4. Results and Discussion

### 4.1. Mechanical Properties

**Figure 3:**
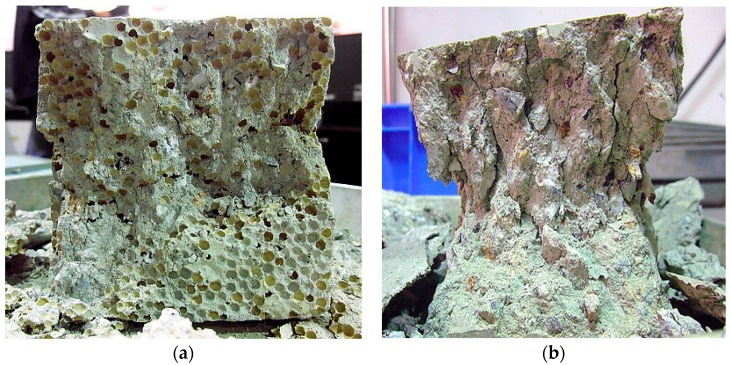
Failure mode of concrete cubes under compressive strength test after exposure to 800 °C: (**a**) PA50; (**b**) Control concrete.

The mechanical properties of control (C) and two PAC mixes (PA25 and PA50) are presented against temperature and polystyrene aggregate content in Table 2. The results show the compressive strength of PA50 is almost half of that of PA25, similar to the compressive strength ratio between PA25 and C. This descending trend in the compressive strength has been associated with the higher degree of air voids, lower density and weak bond between cement paste and polystyrene aggregate [9,25,26]. A decreasing trend is also evident for tensile strength by increasing the percentage of polystyrene aggregate. Figure 3 compares the failure mode of a PAC concrete cube and a control concrete cube under compressive strength test after exposure to 800 °C. It can be seen that all polystyrene beads in the PAC cube melted. Apparently, the control concrete showed a more brittle failure than the PAC concrete cube.

The residual compressive strengths of C and PAC mixes increased after heating up to 150 °C and then decreased rapidly at higher temperatures. Similar results were found in other structural lightweight concrete containing fly ash [27]. Castillo and Durrani explained that the strength gains at temperature below 200 °C are mainly attributed to the increase in the forces between gel particles (Van der Waals forces) owing to the removal of water content [28]. Overall, tensile strength decreases with increasing the temperature from 25 °C to 800 °C except for control concrete whose tensile strength rises up to almost 14% of its original value at 25 °C when the temperature grows to 150 °C. The compressive strength of C, PA25 and PA50 reaches the highest value at 150 °C with 1%, 14% and 11% higher than their corresponding strength values at 25 °C respectively, followed by a decreasing trend until 800 °C. The observed overall decreasing trend supports the results reported in the past studies [29,30,31,32].

**Figure 4:**
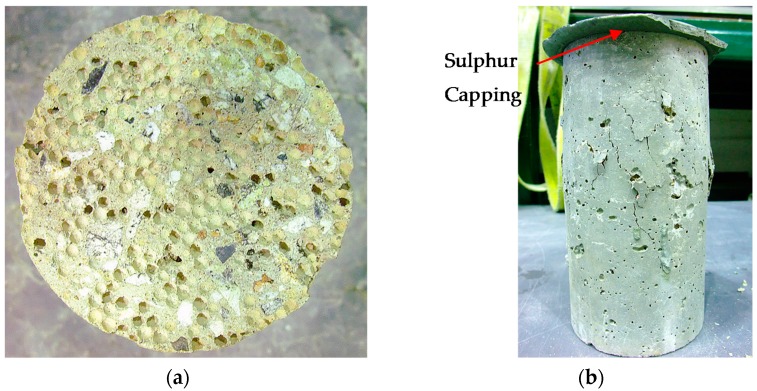
(**a**) PA50 cylinder surface after exposed to 800 °C; (**b**) Failure mode of PAC cylinder (exposed to 800 °C) after test of elastic modulus.

Table 2 also shows the results of modulus of elasticity of PAC. The elastic modulus of concrete is closely dependent on the property of the cement paste, the stiffness of the selected aggregates, and also the method of determining the modulus. Figure 4 shows the failure mode of a concrete cylinder after elastic modulus test. In the current study due to the exposure to high temperature, the top surface of the cylinder was too rough due to the melting of polystyrene beads. Therefore, a thin cement paste followed by a sulphur capping was used to make a flat surface before the modulus of elasticity test.

Comparing the elastic modulus values obtained for C, PA25 and PA50 reveals that replacing each 25% of the normal aggregate with polystyrene aggregate lead to almost 30% to 50% reduction in modulus of elasticity depending on the test temperature. The lower stiffness of the polystyrene aggregate compared to the normal aggregate explains this manner. Moreover, a negative correlation is evident between temperature and the elastic modulus. Increasing temperature to 150 °C, caused about 2% decrease in modulus of elasticity for control concrete while elastic modulus of PA25 and PA50 showed respectively near 18% and 22% reduction as the result of the same temperature increase. Concurrent reduction of the modulus of elasticity and the compressive strength, pointed out in the preceding researches [33,34], is substantiated here as well.

**Figure 5:**
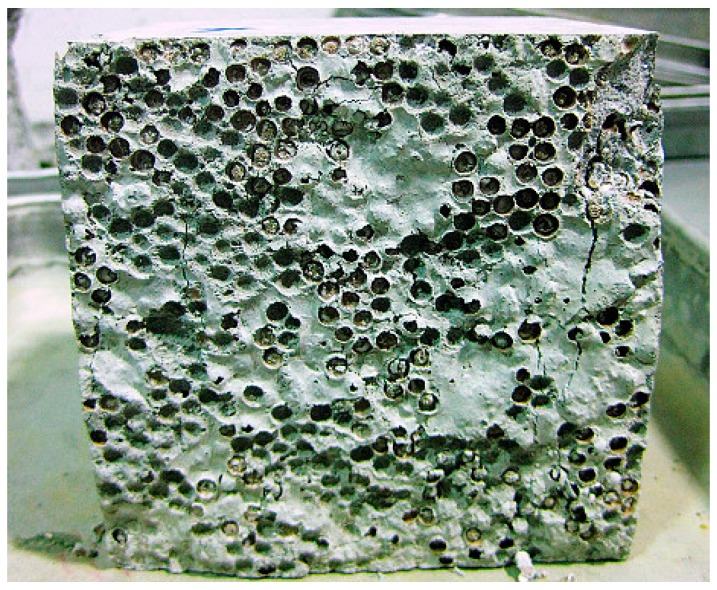
PA50 cube after exposure to 150 °C.

The results presented in Table 2 shows that samples with higher content of polystyrene aggregate are more sensitive to temperature changes. For instance, at 800 °C, compressive strength of PA50 and PA25 is respectively 27.8% and 37.5% of their compressive strength at room temperature, while for control concrete this percentage is equal to 44.7%. The same trend is visible for other mechanical properties at any temperature higher than 150 °C. One possible explanation for this behaviour is based on polystyrene aggregate behaviour at high temperature. They normally start to melt at 100 °C and depending on exposure time and existence of oxygen start to ignite at temperature around 400 °C [35]. The fumes generated from their ignition exert additional internal pressure weakening the PAC compared to the control concrete. Figure 5 shows the melted PA on the surface of PA50 cube after exposure to the temperature of 150 °C.

### 4.2. Fracture Properties

**Figure 6:**
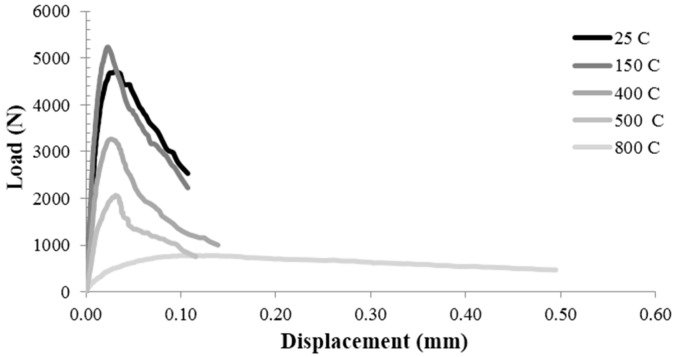
The typical load-deflection curves at five different temperatures for control concrete.

**Figure 7:**
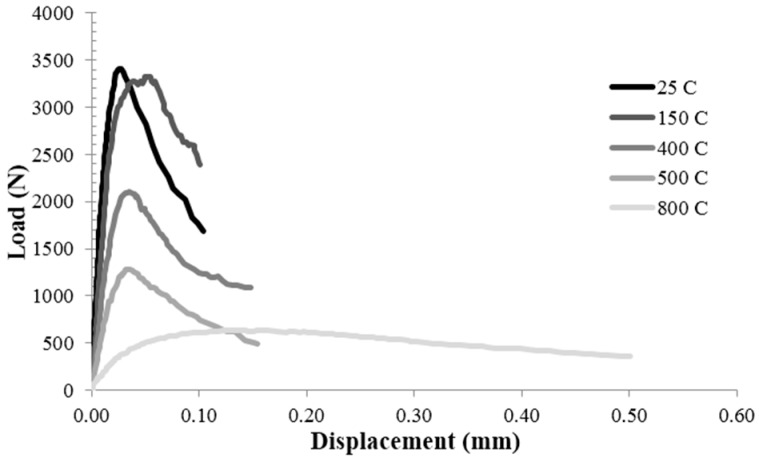
The typical load-deflection curves at five different temperatures for PA25.

**Figure 8:**
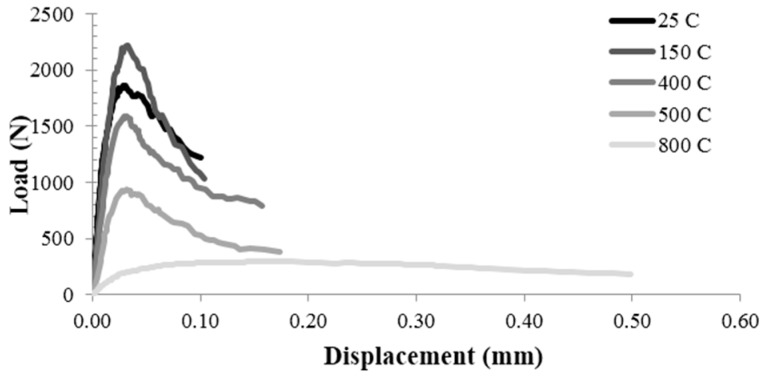
The typical load-deflection curves at five different temperatures for PA50.

The fracture properties of C, PA25 and PA50 were investigated by three-point bending specimens with the same size at varying temperature and polystyrene aggregate content. The typical load-deflection curves at five different temperatures, for control concrete, PA25 and PA50, are presented in Figure 6, Figure 7 and Figure 8 respectively. The curves clearly could be divided into three parts. The first part is the pre peak linear segment which represents the stiffness of the specimen. Figure 6, Figure 7 and Figure 8 show that at higher temperature this parts become less steep which means lower amount of elastic modulus. The second part is the distance between the point where the curve become nonlinear and the peak load. This segment depicts the stable crack growth in the fracture process zone (FPZ). As the temperature rises, this amount grows which shows the larger numbers of micro cracks before peak load. This could be described by the weaker aggregate-cement paste bond at higher temperatures [31]. The third part is the post peak segment which is known as the softening behaviour of the specimen. Increasing the temperature from 25 °C to 500 °C caused a slight change in the post peak behaviour so that at higher temperature the curves drop a little more smoothly. However, this effect is highly dramatic at 800 °C.

Detailed information regarding the fracture properties of control concrete, PA25 and PA50 are provided in Table 3. The table contains the quantity of the critical effective crack length ratio ($a_{c}/d$), critical stress intensity factor ($K_{IC}^{S}$), critical crack tip opening displacement ($CTOD_{C}$), critical strain energy release rate ($G_{IC}^{S}$), and material length (Q), all of which are calculated according to the two-parameter model, and the fracture energy ($G_{F}$), and characteristic length ($l_{ch}$), obtained utilizing fictitious crack model.

#### 4.2.1. Critical Effective Crack Length Ratio ($a_{c}/d$)

The obtained critical effective crack length ratio exhibits erratic behaviour with temperature and polystyrene aggregate content. However, the highest value of $a_{c}/d$ happened at 800 °C for all three samples which was equal to 0.762, 0.668 and 0.771 for control concrete, PA25 and PA50 respectively. These amount were 1.5 times $a_{c}/d$ at room temperature for all samples. The critical effective crack length can be considered as the extension of the fracture process zone (FPZ) before peak load, at which the crack propagation is unstable (i.e. no extra load is required for crack to grow). That is, a larger value of this parameter means a larger FPZ, leading to more ductile behaviour. Thus, it could be concluded that all three concretes show more ductile fracture behaviour at 800 °C.

#### 4.2.2. Critical Stress Intensity Factor ($K_{IC}^{S}$)

**Figure 9:**
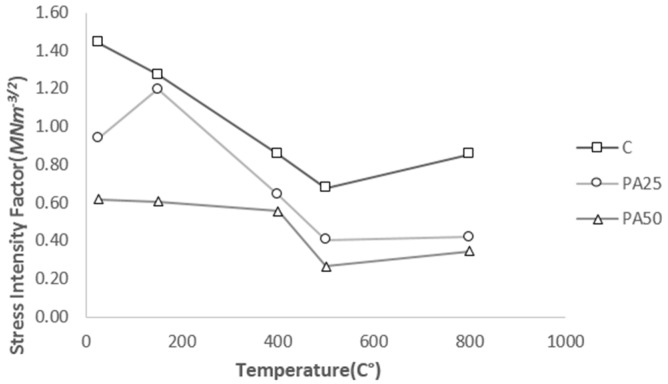
The critical stress intensity factor for three samples at different temperatures.

The stress intensity factor is used to predict the stress state near the crack tip. The critical value of this factor is used to evaluate the toughness of the cracked specimen under load. The higher the $K_{IC}^{S}$ is the tougher the material behaves. The iteration method was employed to determine this factor. Figure 9 compares the critical stress intensity factor of three samples.

The obtained values for C are consistent with those published in literatures [33]. It can be seen from Table 3 and Figure 9 that the values of critical stress intensity factor decrease at higher level of temperature and polystyrene aggregate content. At 800 °C, after a reduction of almost 50% compared to its amount at 25 °C, $K_{IC}^{S}$ reached 0.855, 0.419 and 0.345 MNm^−3/2^ for control concrete, PA25 and PA50 respectively. However, the values of $K_{IC}^{S}$ for all three concretes show an upward trend from 500 °C to 800 °C. Weakening of the cement paste-aggregate bond might be the main reason behind the reduction of $K_{IC}^{S}$ at higher temperature [22]. Comparing the quantities of $K_{IC}^{S}$ at the same temperature for three samples shows that replacing more normal aggregate with polystyrene aggregate leads to a smaller value of $K_{IC}^{S}$. This could be due to the lower load bearing capacity of PAC at crack initiation, corresponding to lower compressive strength of PAC compared to control concrete. This is in good accordance with reports by Gettu et al. [36] and Shah [37] on increasing $K_{IC}^{S}$ with increasing compressive strength.

#### 4.2.3. Critical Crack Tip Opening Displacement ($CTOD_{C}$)

As it can be seen from Table 3, there is a considerable data scattering with respect to $CTOD_{C}$ for all three concretes. Such dispersal could be the result of the method used for unloading compliance estimation. However, higher values were obtained for the PAC specimens compared to control concrete. This could be ascribed to the higher porosity and lower strength of PAC compared to control concrete. Although no clear relationship between temperature and $CTOD_{C}$ was found, considerably larger amount of $CTOD_{C}$ was obtained at 800 °C compared to the ones at room temperature. So that the quantities equal to 8.00, 7.00 and 8.00 mm obtained for $CTOD_{C}$ at 25 °C increased to 23.00, 18.00 and 19.00 mm for C, PA25 and PA50 respectively.

#### 4.2.4. Critical Strain Energy Release Rate ($G_{IC}^{S}$)

The critical strain energy release rate is the required energy for crack to initiate. Similar to the trend in $K_{IC}^{S}$, calculated quantities for $G_{IC}^{S}$ drop by rising the polystyrene aggregate percentage. Change in $G_{IC}^{S}$ with temperature indicates to be an unpredictable function of polystyrene aggregate content at least until 500 °C. Increasing temperature from 25 °C to 500 °C leads to more than 50% reduction in $G_{IC}^{S}$ for control concrete. This temperature variation affects PA25 and PA50 differently as it resulted in fluctuations of $G_{IC}^{S}$ between 18.5 and 70 Nm/m^2^ for PA25 and between 11.7 and 27 Nm/m^2^ for PA50. When temperature changes from 500 °C to 800 °C, this parameter increases dramatically for all samples reaching 65.0, 53.0 and 34.1 Nm/m^2^ for C, PA25 and PA50 respectively.

#### 4.2.5. Material Brittleness

The characteristic length ($l_{ch}$) of the fictitious crack model, along with the material length (*Q*) of the two-parameter model were calculated to study the brittleness of materials. The characteristic length can be described as the length of fracture process zone, while the material length has no direct physical interpretation. The larger the value of these factors, the less brittle the material response to loading. According to Table 3, the characteristic length calculated for control concrete tested in a temperature range from 25 °C to 800 °C takes a value between 200 to 500 mm. This result is in good agreement with that by Karihaloo’s findings [38]. Increasing the proportion of polystyrene aggregate resulted in a higher value of characteristic length, corresponding to less brittle material. These findings are in compliance with the results obtained by Trussoni [26]. Following the trend in critical crack tip opening displacement, all three concretes reach the highest value of $l_{ch}$, equal to 397, 743 and 637 mm for C, PA25 and PA50 respectively, at 800 °C. The material length variation with temperature and aggregate content appeared to be erratic. However, a general increasing trend can be noticed as polystyrene content goes up.

#### 4.2.6. Fracture Energy ($G_{F}$)

**Figure 10:**
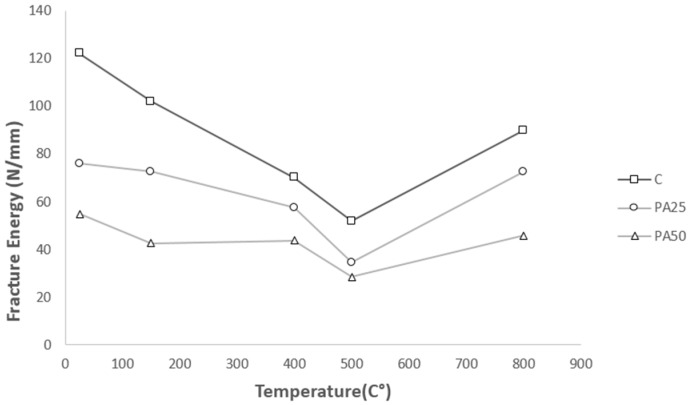
The Fracture Energy for three samples at different temperatures.

Figure 10 shows that the quantities of fracture energy diminish by rising the temperature from 25 °C to 500 °C for all three concretes, supporting the trend reported by Bazant [14]. Meeting their lowest values, the amounts of fracture energy for control concrete, PA25 and PA50 reach 51.9, 34.5 and 28.5 Nm/m^2^ respectively at 500 °C. Increasing temperature to 800 °C, the fracture energy of samples begins to grow.

Comparing the fracture energy at very high temperature, namely 800 °C, with that at room temperature for all three concretes, it is clear that PAC samples perform better in residual fracture energy so that at 800 °C fracture energy of the control concrete declines to near 60% of its original value at room temperature while both PAC samples show less than 20% reduction in fracture energy compared to the room temperature. The results, presented in Table 3, show that by increasing the content of polystyrene aggregate the amount of fracture energy drops appreciably. The reason is behind the way that polystyrene aggregate content influences peak load and post-peak samples’ deflection. In fact, increasing the polystyrene aggregate content enhances the deflection capacity of the samples. However, it dramatically decreases the restricted area by the load-COMD curve due to lessening the peak load magnitude. In a microscopic point of view, it could be explained by the fact that polystyrene aggregates tend to bond loosely to cement paste due to their smoother surface [25]. On the other hand, as it was mentioned previously, low melting and ignition point of polystyrene aggregate may produce fumes causing internal pressure which weaken the PAC samples compared to control concrete. As the result, much less energy is required to cause failure in the PAC samples compared to control concrete.

## 5. Conclusions

In this study the effect of polystyrene aggregate and elevated temperatures on the mechanical and fracture properties of concrete were investigated experimentally. Two samples with 25% and 50% polystyrene aggregate content and a sample of control concrete were studied using three-point bending tests according to RILEM recommendations. Following are the main findings of the current research:

1. Compressive strength, tensile strength and elastic modulus of both PAC samples and the control concrete also reduced under the effect of increasing temperature. However, mechanical properties of PAC samples showed marginally more temperature sensitivity than control concrete.
2. The critical stress intensity factor, the fracture energy and the critical strain energy release rate decreased by increasing the polystyrene aggregate content. It means that less energy is required for crack initiation and crack propagation in PAC samples compared to control concrete. This could be explained by lower aggregate-cement paste bond in PAC in comparison to control concrete.
3. It was observed that PAC samples have higher values of characteristic length. This means replacing more normal aggregate with polystyrene aggregate caused the material failure mode to be more ductile.
4. Increasing temperature affects the fracture properties of all three concretes similarly. Elevating the temperature from 25 °C to 500 °C reduced the amount of $K_{IC}^{S}$, $G_{F}$ and $G_{IC}^{S}$, leading to less cracking resistance in all specimen. In contrast, changing the temperature from 500 °C to 800 °C caused an increase in the required cracking energy.

## Figures and Tables

**Table 1:** Mix details of concrete tested.

Mix Code	Cement (kg/m^3^)	10 mm Aggregate (kg/m^3^)	Sand (kg/m^3^)	Water (kg/m^3^)	Bulk Volume of PA in Liters (V)	Absolute Vol. Fraction of PA in Mix (%) *	Weight of PA Used (kg)
NC	390	1130	610	195	-	-	-
PA25	390	850	610	195	190	12.1	4.56
PA50	390	565	610	195	380	24.2	9.12

* The bulk volume (V) of PA in the mix is determined by dividing the absolute volume fraction with a factor of 0.64.

**Table 2:** Residual mechanical and fracture properties of concrete specimens after exposure to different elevated temperature.

CONCRETE MIX	Peak Temp	Density kg/m^3^	Mechanical Properties							Fracture Properties	
			*f*_c_		*f*_t_		*f*_t_*/f*_c_	*E*		*G_F_*	
			N/mm^2^	%	N/mm^2^	%		KN/mm^2^	%	N/m	%
C	25 °C	2325	56.8	100.0	4.30	100.0	7.57	37.3	100.0	122.2	100.00
	150 °C	2325	57.7	101.6	4.90	114.0	8.49	36.7	98.4	102.1	83.53
	400 °C	2325	45.2	79.6	3.35	77.9	7.41	21.7	58.2	70.1	57.38
	500 °C	2325	34.3	60.4	2.50	58.1	7.29	17.7	47.5	51.9	42.48
	800 °C	2325	25.4	44.7	1.30	30.2	5.12	7.46	20.0	90.0	73.65
PA25	25 °C	2050	26.4	100.0	2.90	100.0	10.98	25.1	100.0	76.0	100.00
	150 °C	2050	30.1	114.0	2.80	96.6	9.30	20.4	81.3	72.6	95.52
	400 °C	2050	20.4	77.3	1.90	65.5	9.31	14.2	56.6	57.5	75.66
	500 °C	2050	15.2	57.6	1.35	46.6	8.88	8.9	35.5	34.5	45.32
	800 °C	2050	9.9	37.5	0.70	24.1	7.07	5.02	20.0	72.6	95.46
PA50	25 °C	1770	13.3	100.0	1.50	100.0	11.28	17.4	100.0	54.9	100.00
	150 °C	1770	14.8	111.3	1.40	93.3	9.46	13.5	77.6	42.6	77.51
	400 °C	1770	9.2	69.2	0.95	63.3	10.33	8.8	50.6	43.8	79.66
	500 °C	1770	6.9	51.9	0.75	50.0	10.87	6.1	35.1	28.5	51.92
	800 °C	1770	3.7	27.8	0.50	33.3	13.51	3.48	20.0	45.8	83.33

**Table 3:** Fracture properties of concrete specimens after exposure to different elevated temperature (*a*_0_/*d* = 0.267).

Mix Code	Temp °C	$a_{c}/d$	$K_{IC}^{S}$ (MNm^−3/2^)	*CTOD_C_* (mm), 10^−3^	$G_{IC}^{S}$ (Nm/m^2^)	*Q* (m), 10^−2^	$G_{F}$ (Nm/m^2^)	$l_{ch}$ (m)
	25	0.46	1.44	8.00	55.8	4.40	122.2	0.246
	150	0.378	1.27	6.00	44.2	3.00	102.1	0.156
C	400	0.405	0.86	7.00	33.7	3.50	70.1	0.136
	500	0.48	0.68	8.00	26.0	4.60	51.9	0.147
	800	0.762	0.85	23.00	65.0	3.90	90.0	0.397
	25	0.423	0.94	7.00	35.2	3.80	76.0	0.227
	150	0.512	1.20	13.00	70.3	4.90	72.6	0.189
PA25	400	0.458	0.64	9.00	29.1	4.30	57.5	0.226
	500	0.468	0.41	10.00	18.5	4.40	34.5	0.168
	800	0.668	0.42	18.00	53.0	4.90	72.6	0.743
	25	0.486	0.62	8.00	22.1	4.60	54.9	0.425
	150	0.419	0.61	9.00	27.2	3.80	42.6	0.293
PA50	400	0.501	0.56	14.00	35.1	4.80	43.8	0.427
	500	0.433	0.27	9.00	11.7	3.90	28.5	0.309
	800	0.771	0.35	19.00	34.1	3.60	45.8	0.637
